# Fertility preservation in male cancer patients. Counseling and reproductive outcomes

**DOI:** 10.3389/fcell.2023.1240152

**Published:** 2023-08-16

**Authors:** Dana Kimelman, Andrea Torrens, Carla Bonelli, Rossana Sapiro

**Affiliations:** ^1^ Oncofertility Program, Centro Hospitalario Pereira Rossell, Administración de los Servicios de Salud del Estado (ASSE), Montevideo, Uruguay; ^2^ Reprovita Lab and Biobank, Montevideo, Uruguay; ^3^ Clínica Ginecotocológica “B”, Facultad de Medicina, Universidad de la República, Montevideo, Uruguay; ^4^ Unidad Académica Histologia y Embriologia, Facultad de Medicina, Universidad de la República, Montevideo, Uruguay

**Keywords:** fertility preservation, male infertility, male cancer, reproductive outcomes, counseling

## Abstract

**Introduction:** Advances in cancer treatments have determined an increase in survival rates. However, these lifesaving therapies may have a negative impact on reproductive health. To diminish the infertility risk; different fertility preservation strategies have been designed. Sperm freezing is the gold standard fertility preservation method in the case of post-pubertal men. The main objective of this study is to evaluate the fertility status of Uruguayan male cancer survivors who have gone through sperm freezing, as well as to assess oncofertility counseling received by these patients.

**Methods:** This is a descriptive, cross-sectional, observational, and transversal study. A survey was conducted on male cancer survivors who cryopreserved sperm between 1985 and 2021 in “Reprovita Lab and Biobank” which is the only sperm bank in this country.

**Results:** One hundred thirty-five participants answered the survey. At the time of diagnosis, the mean age of patients was 28.8 ± 6.4 years old. Testicular was the most frequent type of cancer (64%). Only, 12% (*n* = 15) already had children at the time of diagnosis. Among the interviewed survivors, 50% (*n* = 62) attempted to conceive after cancer treatment, and 68% (*n* = 42) achieved natural pregnancy. Patients who did not achieve spontaneous conception (*n* = 11), used their cryopreserved samples, and 45.4% achieved pregnancy. About 86% (*n* = 107) of survivors believed that the timing of oncofertility referrals was appropriate and 97% considered that having the possibility of protecting their fertility was very important. Eighty percent (*n* = 101), were advised by their attending physicians, 14% (*n* = 18) sought advice from family members or friends, and 4% (*n* = 5) from oncofertility specialists.

**Discussion:** To our knowledge, this is the first study evaluating the reproductive outcomes of male cancer survivors in our country and the region. Most of the interviewed survivors considered fertility preservation as a positive initiative, independent of their reproductive outcomes, reflecting the importance of fertility preservation counseling as one of the most important aspects for futurequality of life of young cancer patients.

## 1 Introduction

Six percent of male cancer diagnoses in Uruguay occur in patients who are under the age of 40 (Incidencia cancer, 2019). Advances in cancer diagnosis and treatments have improved the survival rates of this population. However, these treatments may have a negative impact on future fertility affecting the quality of life of young cancer survivors (Kohler et al., 2011; Alsharhrani et al., 2017). For this reason, health providers should focus their attention on the quality-of-life aspects, which are usually as relevant as the disease for survivors (Mulder et al., 2021). Cancer treatments may affect future fertility in different ways. Drugs have different grades of gonadotoxicity and this also depends on doses, age of the patient, and previous fertility status (Alsharhrani et al., 2017; Ono et al., 2022). Gonadotoxic risks are cataloged as high, moderate, low, and unknown risks (El Issaoui et al., 2016). The toxicity of radiotherapy depends on the doses of radiation, and the target area, low doses of radiation as 0.1 and 1.2 Gy may negatively impact spermatogenesis, and doses over 4 Gy may cause permanent azoospermia (De Felice et al., 2019).

In some cases, spermatogenesis may be affected temporally after treatment and there may be recovery of the function that may take years depending on the treatment and the patient’s age. Sperm cryopreservation should be done ideally before initiating cancer treatments (El Issaoui et al., 2016).

In order to diminish the infertility risk; different fertility preservation strategies have been designed. Sperm, egg, and embryo freezing are some of the available fertility preservation techniques. International guidelines (Practice Committee of the American Society for Reproductive Medicine, 2019; Ono et al., 2022) recommend that every young patient with a cancer diagnosis should receive complete oncofertility counseling before treatment initiation (Lambertini et al., 2020). For patients that had been through puberty, gamete cryopreservation (sperm or oocytes) should be offered (Lambertini et al., 2020). In Uruguay, physicians guide their clinical practice on international guidelines when counseling young cancer patients but there are no national guidelines, national registries, or reports that had evaluated the success rates of fertility preservation strategies or their use. Within male fertility preservation strategies, whenever possible, gamete cryopreservation is the standard and preferred technique. As it is recommended; sperm cryopreservation should be performed prior to starting oncologic treatments. This is why timely referrals from the treating medical team are so relevant. The strategy of testicular tissue cryopreservation and potential re-implantation is only performed in an experimental framework (Eugeni et al., 2022) and there is no experience in our country.

Reprovita Lab and Biobank laboratory is a private center specialized in human reproduction. Gamete and embryo cryopreservation are some of the services provided by this institution. Reprovita is the only sperm bank in our country; therefore, all male gametes cryopreserved for oncological reasons are stored there.

The main objective of this work is to know whether male cancer survivors who underwent sperm cryopreservation achieved their reproductive goals. Secondary outcomes are to evaluate the fertility status of patients that cryopreserved semen samples before cancer treatment, to know how frequently cryopreserved specimens had been used, and to investigate whether patients who underwent sperm cryopreservation are satisfied with the reproductive counseling they received prior to starting oncological treatment.

## 2 Materials and methods

### 2.1 Study population

This descriptive and cross-sectional study includes all male cancer patients who cryopreserved semen samples in a single country at the only sperm bank available in the period from 1985 to 2021. Inclusion and exclusion criteria were established. Inclusion criteria: male patients between 15 and 50 years of age at the time of cryopreservation, who cryopreserved gametes in Reprovita sperm bank due to oncological reasons, and who have given their informed consent to participate in this research. The exclusion criteria established were age <15 and >50 at the time of cryopreservation and/or deceased patients. Data collection was done through telephone interviews conducted by the research team. Telephone lines intended for this work were used for the interviews, in order to facilitate and maintain confidentiality. The collected variables are related to patronymic, demographic, and oncological pathology data, oncological treatment, reproductive counseling, reproductive desires, and events. The collected data were coded and registered anonymously.

### 2.2 Procedure and data management

The whole database of the cryobank was reviewed. Out of 2045 male patients who stored semen samples at our bank between 1985 and 2021, 755 of them did so specifically for oncologic reasons so they all were identified as possible participants. Technical laboratory specialists intended to phone call all 755 men through the period from June to September 2022. When the contact was established, the laboratory staff requested their consent to be contacted by the research team. This phone call was recorded.

### 2.3 Semen analyses and cryopreservation procedure

Semen analyses at the time of cryopreservation were requested for the patients included in the study. Sperm concentration, progressive and total motility (progressive and non-progressive motility), vitality, and sperm morphology were evaluated. The sperm analysis was performed based on reference parameters from the World Health Organization (WHO) guidelines of 1999 and 2010, respectively (Alsharhrani et al., 2017; Boitrelle et al., 2021). Concentration and sperm motility post cryopreservation were also evaluated in the 12 patients that attended our clinic for assisted reproductive treatments (ART).

Sperm samples conditioned with cryoprotectant media were cryopreserved at −196°C in liquid nitrogen. Samples were stored in straws with high biological safety freezing, sealed at both ends, and labeled with the patient’s name, identification document number, and processing date. The patient received a report with the characteristics of the cryopreserved sample.

### 2.4 Statistical analysis

Data were entered into a password-protected secure database. All respondents did not reply to all questions, and the missing data were not computed. Data analysis was performed on Version 26 of the IBM SPSS Statistics software package (Armonk, NY). No power calculation was performed as the sample size directly resulted from the number of respondents to the questionnaire.

Categorical variables were presented as percentages. Continuous variables were expressed by arithmetic means and the corresponding standard errors. The normal distribution of the data was tested using the Shapiro-Wilk normality test. Semen parameters previous and post cryopreservation were compared with paired Student’s t-test. The chi-square test was applied to analyze the percentage of men acquiring pregnancy. A *p*-value <0.05 was considered statistically significant.

### 2.5 Ethical considerations

This study has been evaluated and approved by the Institutional Review Board of the School of Medicine at the Universidad de la República (UdelaR).

## 3 Results

### 3.1 Characteristics of the analyzed population

**FIGURE 1:**
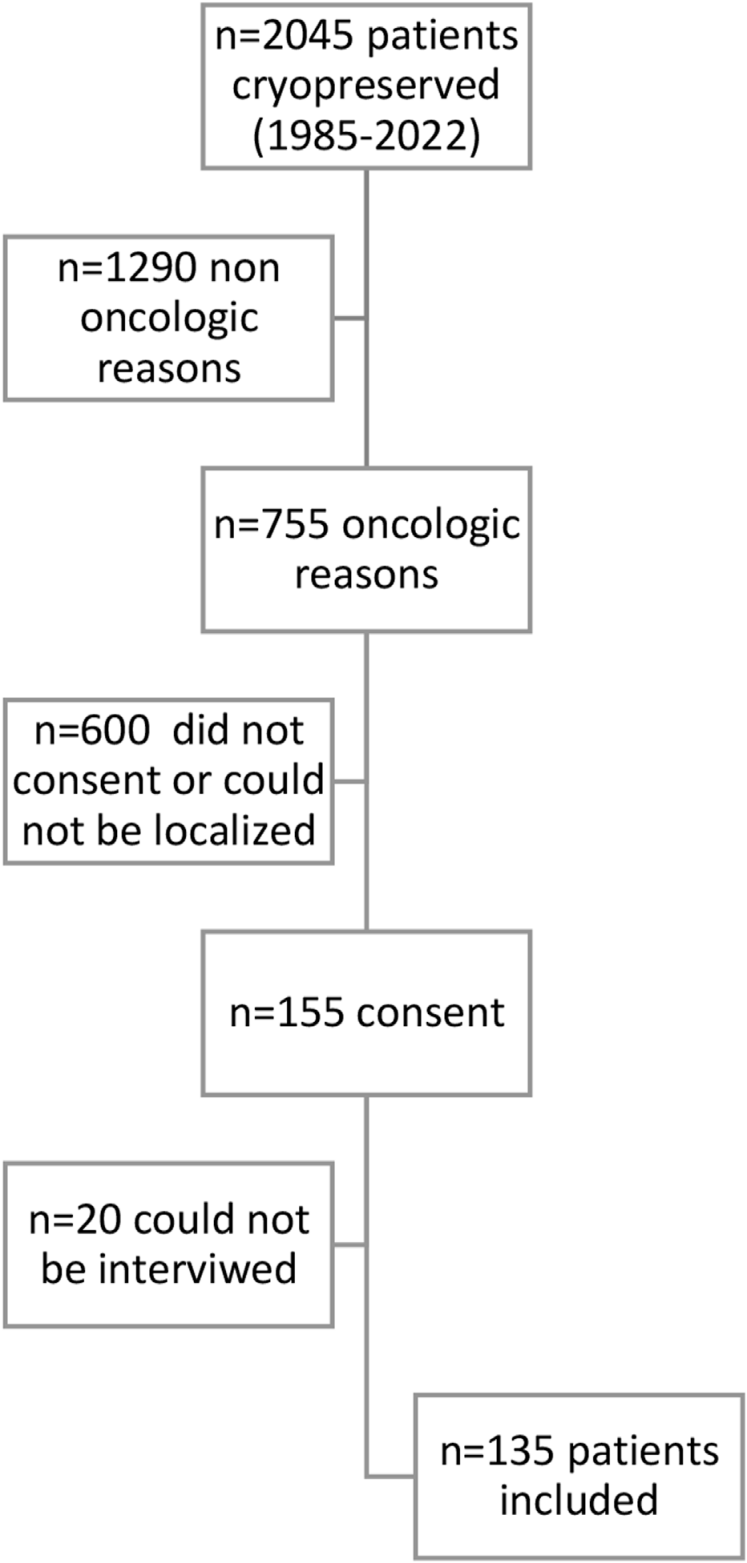
Flow chart of included patients. N=Number of patients included in each step of the analysis.

**FIGURE 2:**
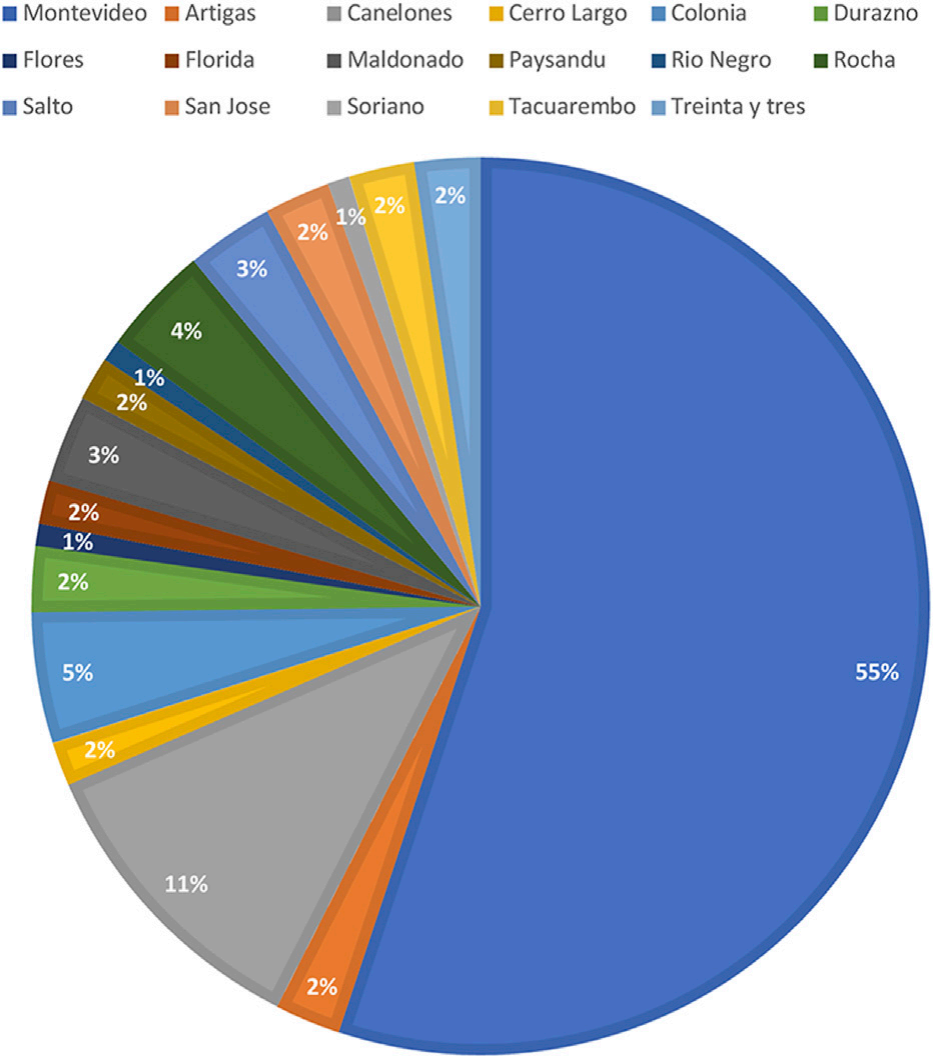
City of residence of surveyed participants. Values are presented as percentages (%).

**FIGURE 3:**
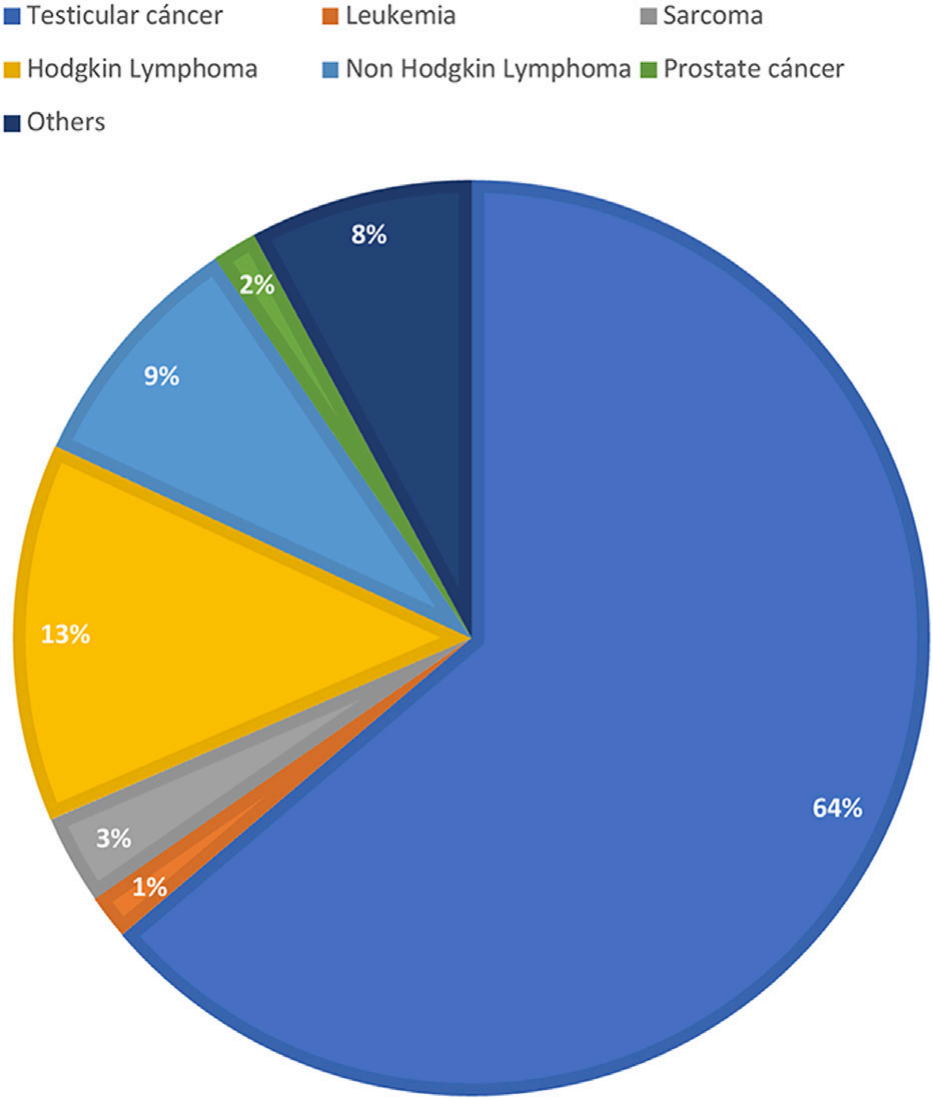
Diagnosis of men that responded the questionnaire. Values are presented as percentasge of patients (%).

Out of the 2045 patients who stored semen samples at our bank between 1985 and 2021, 755 of them did so specifically for oncologic reasons (Figure 1). Only 155 patients (20%) accepted to participate in the study through a survey that was performed through a second call (Figure 1). Different reasons for not participating were: patients who could not be localized, patients who did not answer the phone calls, and patients who did not consent. The recruitment was done through personal phone calls, so patients who died during the period were not registered. Finally, 135 participants that fitted the inclusion criteria answered the questionnaire and were included in the study. Of these patients, 55.1% (*n* = 70) resided in the capital city (Figure 2). The mean age of patients who underwent cryopreservation was 28.8 ± 6.4 years old, whereas the current mean age of this population is 38.1 ± 7.7 years old (Table 1). Among these patients, 64% (*n* = 81) cryopreserved sperm samples due to a testicular cancer diagnosis, while 24% (*n* = 30) had hemato-oncological diseases (Figure 3).

**TABLE 1:** Demographic characteristics of participants. The values are Mean ± SD.

Age at time of survey (mean ± SD)	38.1 ± 7.7
Age at diagnosis (Mean ± SD)	28.8 ± 6.4
City of residence (%)	
Montevideo	55 (n = 70)
Others	45 (n = 57)
Financial coverage for Fertility preservation (%)	
Yes	18.4 (n = 23)
No	80 (n = 100)
Parenthood before cancer diagnosis (%)	
Yes	11.9 (n = 15)
No	88.1 (n = 111)

### 3.2 Reproductive outcomes

When analyzing reproductive outcomes of the interviewed population, our findings showed that at the time of diagnosis, 88% (N = 111) of the patients did not have children, whereas 12% (*n* = 15) already had children (Table 1). Among the interviewees, 56% (*n* = 76) attempted to conceive. All of them had been counseled on not seeking pregnancy before 6 months to 1 year after finalizing oncologic treatment. Out of those survivors who tried to conceive after cancer treatment, 55% (*n* = 42) were successful in achieving pregnancy without the need to use cryopreserved sperm samples. When asked how long they had been trying to conceive, we found that out of 39 respondents, 51% (*n* = 20) achieved pregnancy between one and 2 years of unprotected intercourse. 33% (*n* = 13) achieved pregnancy in two to 3 years, and 13% (*n* = 5) after 3 years. When analyzing the results, we found that 69% (*n* = 29) of testicular cancer survivors and 31% (*n* = 13) of other types of cancer survivors who attempted to conceive were able to achieve pregnancy spontaneously (Table 2). Twelve patients tried to achieve pregnancy through assisted reproductive technologies using cryopreserved samples. Among these patients, 46% (*n* = 5) successfully achieved pregnancy, one patient had no motile sperm in the thawed sample. A comparison between testicular cancer survivors and survivors of other types of cancer who used cryopreserved samples revealed that 67% (*n* = 4) of testicular cancer survivors achieved pregnancy, while only 20% (*n* = 1) of survivors of other types of cancer achieved the same outcome. This comparison did not result statistically significant probably due to the low number of patients included (Table 2).

**TABLE 2:** Reproductive outcomes of participants after treatment.

Spontaneous pregnancy n (%)				Pregnancy using cryopreserved samples (ART) n (%)		
	Yes	No	Total	Yes	No	Total
Testicular cancer	29 (67.4%)	14 (32.6%)	43 (100%)	4 (66.7%)	2 (33.3%)	6 (100%)
Other	13 (59.1%)	9 (40.9%)	22 (100%)	1 (20%)	4 (80%)	5 (100%)
Total	42 (64.6) %	23 (35.4%)	65 (100%)	5 (45.5%)	6 (54.5%)	11 (100%)

Chi square test. Differences between groups were not statistically significant. *n* = number of patients, values are no (%).

### 3.3 Sperm usage

Semen analyses at the time of post-cryopreservation were available for 12 of the patients (those who decided to use the cryopreserved sample for reproductive purposes). One of the samples did not have any motile sperm after thawing. The results of the remaining eleven semen analyses are shown in Table 1. Mean sperm concentration ± standard deviation (61.3 ± 56.0 million/mL), progressive (61.4% ± 13.0%), and total sperm motility (72.7% ± 8.9%) of patients who cryopreserved sperm before initiating gonadotoxic therapy were normal. At the time of thawing, the mean ± standard deviation of sperm concentration significantly decreased to 28.1 ± 27.6 million/mL (*p* < 0.05). The progressive motility was 54.4% ± 20.9% and the total motility 68.5% ± 20.8% (Table 3). All patients that intended to use the cryopreserved samples finalized their cancer treatment more than 1 year before.

**TABLE 3:** Semen characteristics at the time of cryopreservation and at the time of use of sample.

Group	Sperm concentration (10^6^/mL)	Progressive motility (%)	Total motility (%)
	Mean ± SD	Mean ± SD	Mean ± SD
Pre-cryopreservation	61.3 ± 56.0	61.4 ± 13.0	72.7 ± 8.9
Post-cryopreservation	28.1 ± 27.6*	54.4 ± 20.9	68.5 ± 20.8

*n* = 11. *p < 0.01, paired Student’s t-test.

### 3.4 Counseling

Out of 124 participants who answered this question; 86% (*n* = 107) believed that the timing of oncofertility referrals was appropriate, while 12% (*n* = 15) would have preferred an earlier referral. However, 97% of patients feel that having the opportunity to pursue fertility preservation strategies was greatly valuable. When asked about who discussed the potential impact of cancer treatments on fertility, most patients 80% (*n* = 101) indicated that their attending physicians had raised this topic. A small percentage, 4% (*n* = 5), received counseling from oncofertility specialists, and 14% (*n* = 18) sought advice from family members or friends. In interviews with testicular cancer survivors, we inquired about the timing of counseling regarding surgical treatment (orchiectomy). It was revealed that 65% (*n* = 49) received counseling after the surgery, while 36% (*n* = 27) were counseled prior to the procedure. All patients were counseled by oncologists not to seek pregnancy until 6 months after treatment. When it came to financial coverage for fertility preservation strategies, 80% of patients (*n* = 100) paid out of pocket for these techniques and the storage of cryopreserved samples, while 18.4% (*n* = 23) received financial support from their health insurance.

## 4 Discussion

Cancer treatments can lead to impaired fertility (Green et al., 2010). International guidelines recommend that patients should receive complete oncofertility counseling soon after diagnosis and during treatment planning so that fertility preservation strategies may be offered and pursued before treatment (Ono et al., 2022). The attending physician should promote the conversation about possible fertility impairment with every young cancer patient early after the diagnosis (Ono et al., 2022). This exchange not only improves the likelihood that the patient will achieve their reproductive goal, but also improves adherence to the proposed treatments and quality of life (Mulder et al., 2021). For our study, 135 male cancer survivors that cryopreserved sperm samples in the context of cancer diagnosis had been interviewed, this population represents 17.9% of the whole number of patients that cryopreserved semen samples due to oncologic reasons. Most of interviewees had testicular cancer (64%). Consistent with our findings, a study published by Kohler et al. (2011) in 2011, the most common type of cancer among patients who decided to cryopreserve semen samples was Non-Hodgkin’s Lymphoma, Hodgkin’s Lymphoma, and Testicular Cancer; being this the most frequent cancer diagnosis of young male patients worldwide (Hayes-Lattin and Nichols, 2009). Testicular cancer is the most common cancer in men between 20 and 40 years old. During the period 2015–2019, 475 patients between 15 and 39 years old had been diagnosed with testicular cancer in our country; this represents 32% of all cases during the same time range (Incidencia cancer, 2019). Most patients were residents of Montevideo City, the capital of Uruguay. Even though almost 50% of the population of the country lives in the capital city; the underlying reasons for why patients from Montevideo were more likely to undergo fertility preservation is multifactorial. The main reason is that the sperm bank is in this city, which makes access easier. This shows a clear difference in access to cryopreservation treatments in patients from the countryside compared to patients from the capital city. It also seems to be more referrals from physicians from the capital city. Our results suggest the need to generate strategies that allow access throughout the national territory so that patients from the countryside do not miss the opportunity to pursue these treatments. Regarding counseling prior to cryopreservation, most interviewees were advised by their treating physician prior to the start of oncospecific treatment.

Regarding financial coverage and costs of gamete cryopreservation, our findings show that most patients did not receive any financial support from the healthcare system. In the study published by Lackamp et al. in Frontiers Oncology it is indicated that costs may affect cryopreservation rates and future sperm usage (Lackamp et al., 2021), in addition to the lack of information and counseling on cryopreservation, the economic component is a barrier when resorting to this strategy. We must mention that there have been legal changes regarding financing gamete cryopreservation treatments for cancer patients. In July 2022, there was a modification to the assisted reproductive technology regulation law (Law number 19.167). This amendment stipulates that the Uruguayan State is now obligated to provide financial coverage for gamete cryopreservation treatments to all post-pubertal cancer patients under 40 years old. This development is significant in terms of reproductive rights, as the cost of preservation can be a barrier to accessing these strategies. Adequate counseling is part of the recommendations of the Clinical Practice Guideline of the American Society of Clinical Oncology (ASCO), which also considers it appropriate before starting treatment, accompanied by a consultation with an oncofertility specialist (Oktay et al., 2018).

Our results show that most interviewees achieved their reproductive desire through spontaneous pregnancy. However, the number of interviewed patients is low, and these results cannot be extrapolated to all cancer patients. Sheth et al. showed similar outcomes in their study published in 2012 (Sheth et al., 2012), of the 249 patients who cryopreserved semen samples between 2002 and 2010, 21 patients (8.4%) used their sample for assisted reproduction treatments. While in another study conducted at Erasmus Medical Center in the Netherlands (van Casteren et al., 2008), it was shown that 7.5% of patients who cryopreserved between 1983 and 2004 used the sample, and 49% achieved pregnancy. In our research, 12 survivors of all participants intended to use their cryopreserved semen samples, and a total of 45.5% achieved pregnancy with it. Therefore, the results obtained were similar in both studies. In the case of patients who did not achieve pregnancy using cryopreserved samples, several factors that may have determined this result, such as the timing of the sample collection relative to the treatment stage, its quality, and female factors, among others.

Most patients with testicular cancer diagnosis did sperm cryopreservation after orchiectomy, it is important to notice that Emmanuel et al. have shown that there is no evidence showing that expedited radical orchiectomy has an oncological benefit (Emmanuel et al., 2021). Also, Moody et al. demonstrated that effective cryopreservation can be achieved within 1 week of initial diagnosis (Moody et al., 2019). Both conclude in their published work that it is critical to recommend cryopreservation to all patients prior to orchiectomy and/or neoadjuvant chemotherapy regardless of cancer staging even if there is a short temporary delay in orchiectomy (Emmanuel et al., 2021).

We have no knowledge of a study of similar characteristics being conducted neither in our country nor in another Latin American country. In 2021 Lackamp et al. published a survey evaluating “Long- Term Experiences of Sperm Cryopreservation in Oncological and Non-Oncological Patients.” This group evaluated respective outcomes related to different treatment protocols and their results showed that 20.7% of all survivors reported to have fathered at least one naturally conceived child after treatment, especially if they had been treated with less or potentially gonadotoxic therapies (Lackamp et al., 2021). Most of the interviewed patients who cryopreserved sperm and tried to conceive in our study did not have impaired fertility, being able to achieve spontaneous pregnancy. This is consistent with what Brydoy et al.( 2005) have published stating that the spermatogenesis function of many patients recovers after cancer treatment. Like us, Nalesnik et al. (2004) published that 64% of testicular cancer survivors have naturally conceived children. A cohort study of 8.670 male cancer survivors from Denmark and Sweden indicated that 8.162 of the survivors experienced spontaneous pregnancies (Stahl et al., 2011). Although our findings are similar to what our colleagues published before, in our study, only 17.88% of the patients who cryopreserved due to cancer diagnosis were included, which is a deficient percentage. This small percentage may not be representative of the whole population, which is one of our study’s major limitations. Due to the size of the sample studied, it is not possible to conclude whether there was a relationship between the type of cancer, or the received treatment and the rates of spontaneous pregnancy. Of all interviewed survivors, 64% had testicular cancer, and the treatment protocol for this group of patients includes etoposide, cisplatin, and bleomycin from one to four cycles. Of testicular cancer survivors, 69% (*n* = 29) could conceive spontaneously after cancer treatment. Due to the low number of participants, we may not conclude that these chemotherapeutic agents are not gonadotoxic; in fact, there is enough evidence showing the gonad toxicity of these mentioned agents (Sheth et al., 2012; Eugeni et al., 2022).

Even though many survivors achieved spontaneous pregnancy; they still consider that being counseled in oncofertility was very valuable. As the international guidelines have established, every reproductive-aged patient with a cancer diagnosis should be advised in oncofertility before cancer treatment, no matter type of cancer, stage, or prognosis (Lambertini et al., 2020; Ono et al., 2022). Counseling will positively impact how patients will face treatment thinking about life after cancer (Practice Committee of the American Society for Reproductive Medicine, 2019; Lambertini et al., 2020; Ono et al., 2022).

Our work provides valuable data. To our knowledge, this is the first study evaluating the reproductive outcomes of male cancer survivors in our country and the region. The main strength of our work is the fact that our country has only one sperm bank, and this allowed us to include some of the patients who underwent sperm cryopreservation treatment due to oncologic reasons. Our study has some important limitations: the first one is that, as previously mentioned, we only had access to 17.9% of the men who cryopreserved semen samples for cancer reasons. This limitation means that it is only possible to make conclusions based on a small percentage of the population. Another area of improvement to be acknowledged is that the study’s design (through personal phone calls) does not distinguish the causes of why some of the patients cannot be localized. Consequently, valuable data is missing, e.g.: how many patients died for oncological reasons. The third main limitation is not having access to the specific cancer treatment. That information was not asked at the time of cryopreservation, and some patients, while being interviewed for this study, did not remember the treatment they received. It should be noted that although patients who cryopreserved their samples are satisfied with the counseling they received, to objectively evaluate the population of young cancer patients in general, a study would be needed that should include patients who did not undergo cryopreservation. It would be important to continue this research in 5–10 years to follow up on patients who cryopreserved but have not yet expressed reproductive desire. Our future objective will be to compare access to fertility preservation strategies of patients using financial coverage, we believe that much more patients will be able to cryopreserve gametes not only because of financial access but also because of better referrals. We will also need to develop better registries in order to follow up with cancer survivors and have better database information on their disease, treatment, and fertility status after cancer treatment.

## Data Availability

The original contributions presented in the study are included in the article/Supplementary Material, further inquiries can be directed to the corresponding authors.
