# Supplementary material for: Fertility preservation in male cancer patients. Counseling and reproductive outcomes
Source: Front Cell Dev Biol. 2023 Aug 16;11:1240152. doi: 10.3389/fcell.2023.1240152 (PMC10468982; doi:10.3389/fcell.2023.1240152)
Supplement: Supplementary file 1 [file Image1.pdf]

Supplementary figure 1:

Cell Phone Survey:

A. General data

1. Age at the time of the survey

2. Educational level

1- Elementary school
2- Incomplete high school
3- Complete High School
4- University

3. State:

1- Montevideo 2- Artigas 3- Canelones 4- Cerro largo 5- Colonia 6- Durazno 7- Flores 8- Florida 9- Lavalleja 10- Maldonado 11- Paysandú 12- Rio Negro 13- Rivera 14- Rocha 15- Salto 16 -San José 17- Soriano 18- Tacuarembó 19- Treinta y Tres

B. Oncological data

1. Type of cancer

1- Testicular
2- Myeloma
3- Sarcoma
4- Hodgkin's lymphoma
5- Non-Hodgkin's lymphoma
7- Prostate
8- Other

2. Institution

1- Public Hospital
2- University Hospital
3- Private Mutual
4- Medical insurance
5- Abroad
6 -Other Hospitals

3. Year in which the diagnosis was made

4. Age at diagnosis

5. Type of treatment

1- chemotherapy (QT)
2- radiotherapy (RT)
3- surgery,
4- QT, RT and surgery,
5- QT and surgery
6- RT and surgery
7 -QT, RT

6. If PQT: how many cycles?

1- <=4
2- between 4 to 6
3- > 6,
4- I don't remember

C. Sperm cryopreservation counseling

1. Who told you about the possibility of sperm cryopreservation?

1- Family doctor
2- Oncofertility specialist
3- Somebody outside the health system (friends, relatives)
4 -I don't remember

2. When were you counseled?

1- prior to the start of treatment,
2- after the start of treatment

3. In the case of testicular cancer

1- before surgery
2- after surgery

4. Was the timing of the counseling appropriate?

1- yes
2- no
3- I would have liked to have been told before
4- not sure

4. Had your health insurance covered your fertility preservation treatment?

1- yes
2- no
3– I don't know, no answer

D. Fertility Status pre-treatment

1. Did you have children before cancer treatment?

1- yes
2- no

2. Spontaneous pregnancies

1- yes
2- no

3. Number of pregnancies

E. Fertility Status post-treatment

1. Attempted spontaneous pregnancy

1- yes
2- not yet
3- Cannot yet

2. Achieved spontaneous pregnancy

1- yes
2- no

3. Time trying to conceive

1- < 1 year
2- 1-3 years
3->3 years
4- Do not remember

4. Number of pregnancies

5. Newborn gestational age

1- preterm
2- term

F. Fertility Status after recovering cryopreserved sample

1. Did you use the cryopreserved samples?

1- yes
2- no

2. How long after the end of cancer treatment?

1- < 1 year
2- 1 to 5 years
3- > 5 years

3. Type of assisted reproduction treatment

1- In vitro fertilization
2- Artificial insemination

4. Did your partner achieved pregnancy?

1- yes
2- no

5. Miscarriage

1- yes
2- no

6. Number of pregnancies
7. Number of term newborn
8. Number of healthy newborn

G. General comments

1. Do you consider gamete cryopreservation as positive?

1- yes
2- no
3- Prefer not to answer

2. Other comments
